# MutSpot: detection of non-coding mutation hotspots in cancer genomes

**DOI:** 10.1038/s41525-020-0133-4

**Published:** 2020-06-05

**Authors:** Yu Amanda Guo, Mei Mei Chang, Anders Jacobsen Skanderup

**Affiliations:** Computational and Systems Biology, Agency for Science Technology and Research, Genome Institute of Singapore, 60 Biopolis Street, Singapore, 138672 Singapore

**Keywords:** Cancer genomics, Genome informatics

## Abstract

Recurrence and clustering of somatic mutations (hotspots) in cancer genomes may indicate positive selection and involvement in tumorigenesis. MutSpot performs genome-wide inference of mutation hotspots in non-coding and regulatory DNA of cancer genomes. MutSpot performs feature selection across hundreds of epigenetic and sequence features followed by estimation of position- and patient-specific background somatic mutation probabilities. MutSpot is user-friendly, works on a standard workstation, and scales to thousands of cancer genomes.

## Introduction

Cancer is a genetic disease arising from (driver) mutations that give cancer cells a selective advantage to proliferate and invade. Early cancer genomics studies have mainly focused on the protein-coding regions of the genome. However, even with thousands of cancer exomes sequenced in the past decade, identification of putative driver mutations in the coding regions has still not saturated in many cancer types^1,2^. Importantly, mutations in the non-coding DNA that constitutes the other 98% of the human genome is even less explored. Tumor whole-genome sequencing is, however, gaining popularity and a recent study of over 2500 tumor whole genomes by the ICGC/TCGA Pan-Cancer Analysis of Whole Genomes Network (PCAWG) estimated that up to 25% of all tumors harbor non-coding driver mutations^3^. There is therefore a pressing need to develop statistical methods that can leverage these large datasets to predict driver mutations in the non-coding DNA.

Current tools designed to identify non-coding drivers are based on mutation recurrence within regulatory elements^4–6^, predicted functional impact of somatic mutations^7^, or a combination of these approaches^8,9^. However, existing methods are designed to explore mutations within defined regulatory regions, such as promoters, enhancers or UTRs, therefore ignoring the rest of the non-coding genome. As such, a typical non-coding cancer driver detection method evaluates less than 5% of the 3 million bases sequenced in a WGS experiment for signs of positive selection. Furthermore, by restricting the analysis to annotated regulatory regions, current tools will miss non-coding drivers that create *de novo* regulatory elements in regions of unannotated DNA. For example, non-coding mutation hotspots upstream of *TAL1* and *LMO1* in T-cell acute lymphoblastic leukemia lead to the formation of de novo MYB binding sites that drives the overexpression of *TAL1* and *LMO1* oncogenes^10,11^. Here, we present MutSpot, an R package that systematically and unbiasedly scans the entire genome for mutation hotspots with statistical evidence of positive selection.

## Results

### Detection of mutation hotspots in gastric cancer genomes

**Fig. 1:**
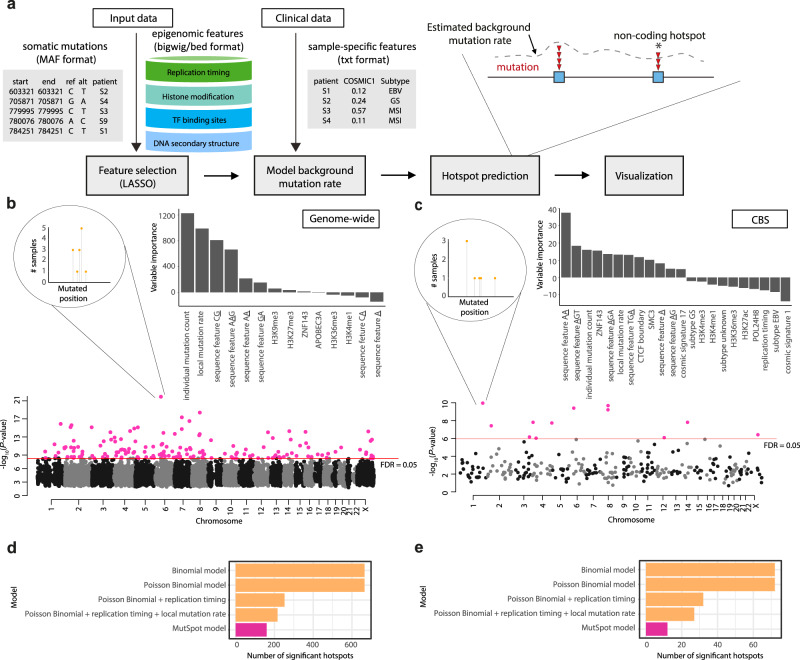
MutSpot analysis on 168 gastric cancer whole genomes. **a** MutSpot analysis workflow. **b**, **c** For each analysis, MutSpot outputs three types of descriptive figures: a Manhattan plot, a feature importance plot of features evaluated by the background mutation model, and lollipop plots of the top hotspots. Figures produced by MutSpot from **b** a genome-wide analysis and **c** a CBS-specific analysis of 168 gastric cancer whole genomes. Hotspots with FDR <0.05 are labeled in magenta. **d**, **e** Comparison of the number of hotspots detected using MutSpot with the number of hotspots detected using other statistical approaches in **d** the genome-wide and **e** CBS-specific analyses.

MutSpot can be used to detect mutation hotspots either genome-wide or in user-defined regions. In the genome-wide discovery mode, MutSpot fits a genomic background model and scans for mutation hotspots across the whole genome. In the regional discovery mode, MutSpot fits a background model specific to the user-defined regions, e.g., promoters, and predicts hotspots in the specified regions only. While the genome-wide mode provides a comprehensive scan of the entire genome, the regional mode can be advantageous when the mutational processes in the regions of interest are very different from the genomic background. To demonstrate the utility of the regional analysis, we ran MutSpot on 168 microsatellite stable gastric cancer whole genomes^12^ to detect SNV hotspots (1) genome-wide and (2) in regions comprising CTCF binding sites (CBS; 47,453 CBSs analyzed). MutSpot identified 160 mutation hotspots genome-wide (2,533,374,732 nucleotides evaluated) and 12 mutation hotspots in CBSs (1,164,231 nucleotides evaluated) at FDR <0.05. In each analysis, MutSpot outputs a Manhattan plot of the detected hotspots and a barplot of the Z-values (quantifying association with mutation rate) of the selected features in the fitted background model (Fig. 1b, c). CBSs are known to be hypermutated in gastrointestinal cancers, with a distinct mutation spectrum enriched in A > G and A > C substitutions^12,13^. In the genome-wide background mutation model, CpG dinucleotides, individual tumor mutation burden and local mutation rate are among the top predictors of mutation probability. In contrast, and consistent with the current knowledge, MutSpot identifies AA dinucleotides as the most important predictor of mutation probability in the CBS-specific model. Twenty-three mutation hotspots at CBSs are identified in the genome-wide model. However, only 12 remain significant in the CBS-specific model that corrects for the elevated background mutation rate and unique mutation spectrum at CBSs.

As there are few validated drivers in the non-coding DNA, we validated the ability of MutSpot to identify known mutation hotspots in the protein-coding regions. MutSpot identified 10 hotspots in four genes using the gastric cancer cohort (Supplementary Fig. 1). All four genes are known drivers of gastric cancer (*TP53*, *CTNNB1*, *KRAS*, and *RHOA*). There were a total of 38 unique protein-altering mutations in the 10 hotspots, and 37/38 mutations are found to be hotspot mutations by a previously published pan-cancer analysis of protein-coding hotspots^2,14^.

### Performance of MutSpot on other tumor cohorts

The statistical power for detection of hotspots depends on factors such as tumor cohort size and the passenger mutation rate in the specific cancer type^1^. To further demonstrate the performance of MutSpot, we ran MutSpot on two additional cancer cohorts with different passenger mutation loads. First, we ran MutSpot on 31 paediatric T-cell acute lymphoblastic leukemia (T-ALL) tumors^11^, using MutSpot default features and lymphocyte-specific epigenetic profiles in the feature selection step (See Supplementary Methods). We identified non-coding hotspots upstream of three known T-ALL oncogenes (*LMO1*, *LMO2*, and *TAL1;* Supplementary Fig. 2), demonstrating that hotspot detection could be useful even in small cancer cohorts. Next, we performed hotspot discovery on 70 melanoma tumors^3^. The high passenger mutation load in melanoma and the presence of local hypermutation at TF-binding sites (TFBS)^15,16^ make hotspot detection in melanoma especially challenging. To account for known mutational biases in melanoma, we included melanoma-specific epigenetic and sequence features in addition to the default MutSpot features for feature selection (See Supplementary Methods). MutSpot identified 79 mutation hotspots at 1% FDR, and the top hotspot identified overlaps the two known hotspot mutations in the *TERT* gene promoter (Supplementary Fig. 3). Melanoma tumors are hypermutated at active TFBSs in gene promoters due to impaired nucleotide excision repair (NER) at these sites^15^. The default MutSpot model without tissue-specific features predicted 104 mutation hotspots with 47 hotspots overlapping gene proximal TFBSs (Supplementary Fig. 3). Using instead a model also correcting for local hypermutation at active TFBSs in melanoma, only 25 out of the 79 significant hotspots identified were located in active TFBSs in gene promoters. By examining common features of the remaining NER-associated hotspots identified by MutSpot, one could potentially identify additional covariates of the somatic mutation processes acting on these sites. Such features could then be modelled by MutSpot in an iterative manner to further refine the background mutation model to reduce false-positive hotspots.

### Comparison to existing methods

We compared MutSpot against other statistical approaches for driver detection adopted by previous studies^17–19^ (Fig. 1d, e, Supplementary Fig. 4). Since none of these approaches are available as standalone software packages for hotspot detection, we implemented four commonly used strategies: (1) Binomial model based on the average genome-wide mutation rate in the cohort, (2) Poisson Binomial model accounting for heterogeneity in genome-wide mutation rates across individual tumors, (3) Poisson Binomial model also correcting for variation in DNA replication timing, and (4) Poisson Binomial correcting for both DNA replication timing and local mutation rate. Expectedly, models that integrated more information about confounding factors predicted fewer candidate hotspots (Fig. 1d, e, Supplementary Fig. 4). Hotspots predicted by only the simpler models are likely false-positives, since their frequency can be explained by genomic covariates of the somatic mutation rate. Overall, this indicates that the larger covariate feature space modelled by MutSpot reduces the number of potential false-positive hits.

A recent study by the PCAWG consortium has examined pan-cancer non-coding drivers using an ensemble of different driver discovery methods^20^. However, most of these methods were designed to identify positive selection in annotated regulatory regions, and none of the methods work out of the box for genome-wide hotspot detection. To further validate the performance of MutSpot, we adapted three existing methods (OncoDriveFML^7^, ncdDetect^4,21^, and ActiveDriverWGS^6^) for genome-wide hotspot detection by first identifying potential hotspot regions (short windows with four or more mutations) and then used these regions as input for each method (see Supplementary Methods). From the cohort of 168 gastric cancer tumors, 87/90 hotspots identified by MutSpot are also found by at least one other method (Supplementary Fig. 5). Similarly, in the cohort of 70 melanoma samples, 74/79 hotspots identified by MutSpot are found by at least one other method (Supplementary Fig. 5). In summary, MutSpot is currently the only standalone tool available for genome-wide identification of mutation hotspots, and the predictions made by MutSpot are generally concordant with other driver identification methods.

## Discussion

MutSpot offers the flexibility to incorporate any genomic or clinical covariate into the background mutation model. This allows users to include tissue-specific epigenetic features for the cancer type of interest, as well as other newly discovered mutational biases into the background mutation model. As our current knowledge of the mutational processes and biases is far from complete, new insights into the processes underlying somatic mutations will further improve the accuracy of hotspot detection.

In conclusion, MutSpot is a user-friendly tool for end-to-end non-coding mutation hotspot identification from cancer genomes. As an increasing number of cancer whole genomes become available, MutSpot can facilitate the discovery of novel drivers in the non-coding genome to further our understanding of tumor biology.

## Methods

### Input features for modeling background mutation rates

Non-coding hotspots are small, focal regions with high recurrence and clustering of somatic mutations. By default, Mutspot defines a hotspot as a 21 bp region with at least two mutations. To accurately detect mutation hotspots, MutSpot builds a logistic regression model to estimate patient- and position-specific background mutation rates while correcting for known covariates of mutation probability, such as local nucleotide context, replication timing, and epigenomic features^12^ (Fig. 1a and Table 1). As mutation hotspot detection can be sensitive to recurrent sequencing or variant-calling artifacts, the users are recommended to prefilter the input mutations to remove likely mapping and sequencing errors (see Supplementary Methods). In addition, MutSpot excludes problematic regions, such as poorly mappable regions and immunoglobin loci, from the analysis. Poorly mappable regions are defined as regions with mappability score <1 in the ENCODE 75mers Alignability track in the UCSC genome browser. Separate background mutation models are built for single nucleotide variants (SNVs) and small insertions and deletions (indels), as they arise from different mutational processes. By default, MutSpot automatically computes 763 sequence, epigenetic, and structural features (Table 1). As replication timing profiles and transcription factor (TF) binding profiles are not yet available for many tissue types, MutSpot provides the mean replication profile of 13 ENCODE cell lines, and the aggregate TF-binding profile over all available ENCODE cell lines as default non-tissue-specific features. However, we expect tissue-specific epigenetic profiles to be more predictive of the background mutation rates in individual cancer types^22^. Therefore, we recommend users to input tissue-specific epigenetic features for feature selection if available. Additional epigenomic features such as DNase I hypersensitive sites (DHSs) and histone modification profiles can be provided by the user in the bigwig or bed format (Table 1). Tissue-specific DHSs and histone modification profiles for a large number of tissues are readily available from the Roadmap Epigenomics Project^23^.

**Table 1:** Details of sequence, epigenetic and structural features that can be included in the MutSpot model.

Feature	Feature detail	Rationale	Source
Sequence context (SNVs)	Identity of mutated base (A/T or C/G). Trinucleotide and penta-nucleotide contexts centered at the mutated base, and 1 bp and 2 bp left and right flanks of the mutated base.	Sequence context is a major covariate of mutation probability. Although previous studies typically considered trinucleotide contexts, mutation rates could be affected by wider sequence contexts^25^.	Computed from mutation data
Sequence context (indels)	Presence of poly-A/T or poly-C/G sequences longer than 5 bp at the indel site.	Long mononucleotide repeats could lead to artifacts in indel calling.	Computed from mutation data
TF-binding profiles	ChIP-Seq peak profiles of 132 TFs and 1 meta profile including peaks of all TFs from ENCODE cell lines.	TF-binding sites have elevated mutation rates in certain cancers due to impaired nucleotide excision repair.	Zerbino et al. ^26^
Replication timing	Mean replication timing profile of 13 ENCODE cell lines.	Replication timing is inversely correlated with mutation probability.	Hansen et al. ^27^
APOBEC editing sites	Predicted APOBEC editing sites.	Elevated mutation rates at APOBEC editing sites could lead to the formation of passenger hotspots.	Buisson et al.^28^ Table S2.
Local mutation rate	Mutation rate of 100 kb nonoverlapping genomic bins.	To correct for additional unexplained regional variation in mutation rates.	Computed from mutation data
Individual mutation count	Mutation burden of individual tumors.	To account for intertumor heterogeneity.	Computed from mutation data
Tissue-specific epigenetic profile	Chromatin accessibility and modification profiles from matched tissue/cell type.	Epigenetic profiles from the cell of origin better predict the mutational landscape of tumors^13^.	Supplied by the user
COSMIC mutation signatures	Proportion of mutations contributed by a specific mutation signature for each tumor.	To further correct for specific mutational processes in the tumor cohort.	Supplied by the user

### Feature selection using LASSO regression

The most predictive features of mutation probabilities are selected by a LASSO logistic regression model. MutSpot randomly samples 1 million mutated sites from the input mutation file (or all mutated sites if the total number of mutations is less than 1 million) and an equal number of non-mutated sites as the input for the LASSO logistic regression model. Then, the mutation status of each site is regressed against all candidate sequence or epigenetic features. The regularization parameter is chosen as the value at which the error of the model is within one standard deviation from the minimum, as determined by 10-fold cross-validation. MutSpot performs LASSO regression on 100 bootstrap samples with 50% of the data in each bootstrap, and selects for epigenomic features with more than 75% recurrence frequency and sequence features with more than 90% recurrence frequency. The user can adjust these thresholds to control the number of features included in the final background mutation model. To determine the number of mutations required for optimal performance of feature selection, we repeated LASSO feature selection on the gastric cancer and melanoma cohorts by sampling 50k, 100k, 250k, 500k, 750k, 1 million (default), 1.5 million, and 2 million mutated sites, and an equal number of non-mutated sites in each experiment. Then, we fitted logistic regression models based on features selected in each experiment, and calculated the MacFadden’s pseudo-R2 to estimate the model fit. We found that the MacFadden’s pseudo-R2 levels off at around 200k sampled sites (100k mutated sites) for both cohorts (Supplementary Fig. 6). Overall, we recommend the tumor cohort to have at least 100,000 mutations for optimal performance of feature selection. MutSpot uses the ‘glmnet’ package for LASSO regression and cross-validation.

### Sample- and position-specific background mutation model

To account for interpatient heterogeneity, MutSpot corrects for the mutation burden of individual tumors. Additional patient-specific features such as mutation signatures and cancer subtypes can also be integrated into the model. Finally, MutSpot fits a logistic regression model over all positions in the genome to estimate patient- and position- specific background mutation probabilities.

1 $${\mathrm{glm}}\left( {y\sim \beta X,{\mathrm{family}} = {\mathrm{logit}}} \right)$$

Here, X includes sequence and epigenetic features selected by LASSO regression as well as sample-specific features such as tumor mutation count and clinical features.

### Identification of mutation hotspots

To identify mutation hotspots, MutSpot evaluates the mutation recurrence for *l*-bp regions with at least *n* mutated samples genome-wide (default *l* *=* 21, *n* = 2). We set the default window size (*l*) to 21 nucleotides because most TF-binding motifs are shorter than 20 nucleotides, and recurrent mutations that create or abolish a specific TFBS should therefore cluster within 20 bp. The user can set the recurrence parameter *n* based on the desired minimum recurrence frequency (e.g., set *n* = 20 for a cohort of 1000 tumors to detect hotspots with at least 2% recurrence). Increasing the recurrence parameter decreases the compute time as fewer regions are evaluated (Supplementary Fig. 7). The p-value of mutation recurrence is computed using a Poisson binomial model that accounts for varying mutation rates across different patient tumors^12,18^. Multiple hypothesis testing is corrected using the Benjamini Hochberg method. Theoretically, MutSpot evaluates each position in the genome and its 20 bp flank for mutation recurrence, although in practice only regions with at least *n* mutated samples are evaluated. Non-evaluated nucleotides with fewer than two mutated samples in its 20 bp flanks are assigned *P* = 1, and *P*-values are corrected for multiple testing across all nucleotides in the masked non-coding genome (2,533,374,732 nucleotides). In the region-specific mode, the number of hypotheses is the number of nucleotides in the masked regions of interest.

As it can be computationally expensive to fit genome-wide models with multiple covariates, sparse matrices were implemented to minimize memory usage and a multi-threading option is available to reduce the compute time. MutSpot takes less than 3 hours on a 4-core machine for genome-wide hotspot discovery in 200 tumors, and it can be scaled up to process thousands of tumors on a standard workstation (Supplementary Fig. 8).

### Preprint

A previous version of this manuscript was published as a preprint^24^.

### Reporting summary

Further information on research design is available in the Nature Research Reporting Summary linked to this article.

## Supplementary information

Supplementary Information

Reporting Summary

## Data Availability

Gastric cancer mutation data are available as Supplementary Data 3 of Guo et al^12^. T-ALL somatic mutations were obtained from Hu et al^11^. Melanoma somatic mutations are available for download at https://xenabrowser.net/. Roadmap Epigenomics data are available for download at http://www.roadmapepigenomics.org/data/. ENCODE data are available for download at ftp://ftp.ensembl.org/pub/release-85/regulation/homo_sapiens/.

## Code availability

MutSpot is implemented as an R package and is available at https://github.com/skandlab/MutSpot/. All R code used to generate the figures and statistics of the paper is included in Supplementary Data.
