# Supplementary material for: MutSpot: detection of non-coding mutation hotspots in cancer genomes
Source: NPJ Genom Med. 2020 Jun 5;5:26. doi: 10.1038/s41525-020-0133-4 (PMC7275039; doi:10.1038/s41525-020-0133-4)
Supplement: Supplementary file 1 — Supplementary Information [file 41525_2020_133_MOESM1_ESM.docx]

**Supplementary Information for**

**MutSpot: detection of non-coding mutation hotspots in cancer genomes**

Yu Amanda Guo^1*^, Mei Mei Chang^1*^ & Anders Jacobsen Skanderup^1^

^1^ Computational and Systems Biology, Agency for Science Technology and Research, Genome Institute of Singapore, 60 Biopolis Street, Singapore 138672, Singapore

*These authors contributed equally to this work

Correspondence should be addressed to Anders Jacobsen Skanderup (skanderupamj@gis.a-star.edu.sg) or Yu Amanda Guo (yg246@gis.a-star.edu.sg)

**Supplementary Methods**

### Input data for MutSpot

MutSpot requires a mutation file in the MAF format, and genomic/epigenomic features in the bigwig or bed formats. Clinical features and other sample specific features can also be supplied in plain text format (optional). MutSpot provides a default set of 135 epigenetic features including replication timing, transcription factor binding profiles and APOBEC editing sites. The user has the option to include additional features such as cancer type specific epigenetic profiles. Discrete epigenetic features such as peak calls of histone modification should be provided in the bed format, and continuous features such as replication timing profile should be provided in the bigwig format. Continuous genomic features will be discretized into 10 equally sized bins, and the mean value of each bin is used for regression.

MutSpot automatically computes sequence features from the input mutation file. Sequence features for SNVs include identity of mutated base (A/T or C/G), the trinucleotide and pentanucleotide context of the mutated site, and the 1bp and 2bp left and right flanks of the mutated site. Sequence features for indels include the presence of mononucleotide repeats of 5bp or longer at the mutated sites. Finally, MutSpot also computes the local mutation rate in 100kb non-overlapping bins to account for additional unknown covariates of regional mutation rates.

**Recommended filtering steps for mutation calls**

As MutSpot detects mutations that cluster within small windows, it is sensitive to recurrent artefacts from sequencing or mapping errors. We recommend the users to apply the following filters to remove potential artefacts from their mutation set:

1. Remove somatic mutations that are found in >10% of the set of matched normal samples. This removes systematic sequencing artefacts that affect both tumor and normal samples.
2. Remove potential germline mutations by excluding mutations found in >1% allele frequency in the normal population (e.g. 1000 genomes project).
3. Remove mutations found in >1% of matched normal samples that are within 20bp from a common germline indel. This removes potential artefacts arising from erroneous mapping near indels.

### Setting the window size and minimum recurrence parameters

We repeated MutSpot genome-wide and CBS-specific analyses on the cohort of 168 gastric tumors varying the window size (*l*) and minimum recurrence (*n*) parameters. Reducing the window size from the default 21bp to 11bp increases the number of hotspots detected, while increasing the window size to 41bp decreases the number of hotspots detected (**Supplementary Figure 7**). This suggests that mutations in most candidate hotspot regions cluster within 11bp. For two regions with the same background mutation rate per nucleotide, the shorter region will have a lower *p*-value than the longer region with the same number of mutated samples. We set 21-bp as the default window to include potential hotspots that affect longer TF-binding motifs (e.g. the CTCF consensus binding motif is 19bp long), and regions where TFs bind as dimers. The users could adjust the window size for their study or run multiple analyses with different window sizes to compare the results. In addition, the compute time in the prediction step increases linearly with the window size as there are more nucleotides to evaluate in longer windows. On the other hand, increasing parameter *n* greatly reduces the number of regions to evaluate and computational time in the prediction step (**Supplementary Figure 7**). In the gastric cancer cohort, increasing n from 2 to 4 does not change the number of predicted hotspots, suggesting that all significant hotspots are mutated in at least 4 tumors. In practice, we suggest the user to set the minimum recurrence parameter based on the cohort size of the study and the desired recurrence level (e.g. set *n*=20 for a cohort of 1000 tumors to detect hotspots with at least 2% recurrence).

**Running MutSpot on gastric cancer, T-ALL, and melanoma tumors**

Gastric cancer: Mutation calls from the whole genome sequencing of 168 microsatellite stable gastric cancer tumors were obtained from Guo et al^1^. Microsatellite instable tumors were excluded from the analysis since these tumors are deficient in mismatch repair and therefore tend to be hypermutated. We performed feature selection on the MutSpot default features and gastric tissue specific DNAse I hypersensitive sites (DHS) and histone modification profiles downloaded from the Roadmap Epigenomics Project^2^. For the CTCF-binding site (CBS) specific analysis, we defined gastric cancer specific CBS as CTCF-binding motifs that overlap both a CTCF ChIP-seq peak in any ENCODE cell line and a DHS in gastric tissue. LASSO stability threshold was set at 1 for sequence features and 0.75 for epigenetic features.

T-ALL: Mutation data from 31 paediatric T-cell acute lymphoblastic leukemia (T-ALL) tumors was obtained from Hu. et al^3^. We applied MutSpot using the default features and DHS and histone modification profiles from lymphocytes downloaded from the Roadmap Epigenomics Project. LASSO stability threshold was set at 0.9 for sequence features and 0.65 for epigenetic features.

Melanoma: Mutation data from 70 melanoma tumors were obtained from PCAWG through the Xena browser^4^. Melanomas have high background mutation rates and are known to be hypermutated at active transcription factor binding sites due to impaired nucleotide excision repair (NER) at these sites^5^. To correct for known mutation biases in melanoma, we added the following additional features for feature selection by MutSpot:

1. Histone modification profiles from melanocytes to correct for melanocyte specific regional variations in mutation rate (downloaded from Roadmap Epigenomics)

2. Binary feature indicating if the site overlaps a CTTCCG motif to correct for context-specific hypermutation^6^.

3. Occupied TF-binding sites in melanoma cell line to correct for hypermutation resulting from impaired NER (downloaded from the supplementary website of Sabarinathan et al.^5^).

Since the 2 known driver mutations in the *TERT* gene promoter are located 22bps apart, we set the window size for hotspot discovery to 31bp for the melanoma cohort. LASSO stability threshold was set at 1 for sequence features and 0.98 for epigenetic features.

**Benchmarking of MutSpot on simulated cancer genomes**

To benchmark the memory and time usage of MutSpot for large tumor cohorts, we simulated SNV profiles of 1000 cancer genomes from whole genome sequencing data of 168 non-hypermutated gastric cancer tumors using the following steps:

1. Identify the 10^th^ (A) and 90^th^ (B) quantiles of tumor mutation burden from 168 gastric cancer whole genomes.
2. Simulate the mutation burden of 1000 genomes by choosing 1000 random numbers (x_1,_ x_2,_ x_3,_ x_4,_ … x_1000_) between A and B.
3. For the i^th^ sample, we sample x_i_ mutations from the 168 gastric cancer genomes. Then for each mutation, we add a random number between -1000 to 1000 to the position to shift the location of the mutation.

We benchmarked MutSpot on 200, 500 and 1000 simulated cancer whole genomes (**Supplementary Figure 8**). We ran MutSpot on the simulated tumours using 4 cores and 6 cores on a R4 machine (8vCPU, 61GiB) on Amazon Web Services. We used the default cutoff for epigenetic feature selection (0.75) and chose the top 5 nucleotide context features for each background model.

**Adapting existing methods for non-coding hotspot discovery**

We compared the performance of MutSpot to 3 existing methods (OncoDriveFML^7^, ActiveDriverWGS^8^, and ncdDetect2^9^) in the gastric cancer and melanoma cohorts. As existing methods are designed to analyse annotated regulatory regions for signs of positive selection, we first defined the input regions by identifying all non-coding genomic windows with at least 4 mutated samples (window size=21 in gastric analysis and 31 in melanoma analysis).

OncoDriveFML was applied using default parameters using CADD^10^ scores as a measure of functional impact. ActiveDriverWGS was applied with default parameters. NcdDetect2 identifies recurrently mutated regions based on sample- and position-specific mutation probabilities. However, since ncdDetect2 does not provide the code to predict sample- and position-specific mutation probabilities for new samples, we used the genomic average mutation rate for each sample to run ncdDetect. We set the overdispersion parameter to 0.266, as estimated by the ncdDetect2 paper. Since ActiveDriverWGS and ncdDetect2 identify candidate driver regions based on mutation recurrence, we performed multiple testing correction in the same way as in MutSpot, where the number of hypothesis tested is the size of the masked non-coding genome.

**References**

1 Guo, Y. A. *et al.* Mutation hotspots at CTCF binding sites coupled to chromosomal instability in gastrointestinal cancers. *Nat Commun* **9**, 1520, doi:10.1038/s41467-018-03828-2 (2018).

2 Roadmap Epigenomics Consortium *et al.* Integrative analysis of 111 reference human epigenomes. *Nature* **518**, 317-330, doi:10.1038/nature14248 (2015).

3 Hu, S. *et al.* Whole-genome noncoding sequence analysis in T-cell acute lymphoblastic leukemia identifies oncogene enhancer mutations. *Blood* **129**, 3264-3268, doi:10.1182/blood-2017-03-771162 (2017).

4 Pan-Cancer Analysis of Whole Genomes Consortium. Pan-cancer analysis of whole genomes. *Nature* **578**, 82-93, doi:10.1038/s41586-020-1969-6 (2020).

5 Sabarinathan, R., Mularoni, L., Deu-Pons, J., Gonzalez-Perez, A. & Lopez-Bigas, N. Nucleotide excision repair is impaired by binding of transcription factors to DNA. *Nature* **532**, 264-267, doi:10.1038/nature17661 (2016).

6 Fredriksson, N. J. *et al.* Recurrent promoter mutations in melanoma are defined by an extended context-specific mutational signature. *PLoS Genet* **13**, e1006773, doi:10.1371/journal.pgen.1006773 (2017).

7 Mularoni, L., Sabarinathan, R., Deu-Pons, J., Gonzalez-Perez, A. & Lopez-Bigas, N. OncodriveFML: a general framework to identify coding and non-coding regions with cancer driver mutations. *Genome Biol* **17**, 128, doi:10.1186/s13059-016-0994-0 (2016).

8 Zhu, H. *et al.* Candidate Cancer Driver Mutations in Distal Regulatory Elements and Long-Range Chromatin Interaction Networks. *Mol Cell*, doi:10.1016/j.molcel.2019.12.027 (2020).

9 Juul, M. *et al.* ncdDetect2: improved models of the site-specific mutation rate in cancer and driver detection with robust significance evaluation. *Bioinformatics* **35**, 189-199, doi:10.1093/bioinformatics/bty511 (2019).

10 Rentzsch, P., Witten, D., Cooper, G. M., Shendure, J. & Kircher, M. CADD: predicting the deleteriousness of variants throughout the human genome. *Nucleic Acids Res* **47**, D886-D894, doi:10.1093/nar/gky1016 (2019).

**Supplementary Figure 1** Performance of MutSpot on coding regions**. (a)** Manhattan plot shows significant hotspots at FDR of 0.05 colored in maroon. MutSpot was run on 168 gastric cancer tumors in coding regions only.

**Supplementary Figure 2** Performance of MutSpot on T-ALL**.** MutSpot was run on 31 T-ALL samples genome-wide. **(a)** Manhattan plot shows significant SNV hotspots at FDR 0.05 colored in maroon. **(b)** Manhattan plot shows significant indel hotspots at FDR 0.05 in maroon.

**Supplementary Figure 3** Performance of MutSpot on melanoma. **(a)** Manhattan plot shows significant hotspots at FDR 0.01 colored in maroon. Maroon triangles represent hotspots that overlap proximal TFBSs in melanoma cell line. MutSpot was run on 70 melanoma samples genome-wide with *min.count*=4 and *hotspot.size*=31. **(b)** Barplot shows the number of hotspots detected by a simple Binomial model, the MutSpot default model and the MutSpot cancer-specific model at FDR 0.01.

**Supplementary Figure 4** Overlaps among hotspots detected by MutSpot and simpler models**. (a)** Venn diagram shows the overlaps among the hotspots detected by models defined in **Figure 1 (d)** at FDR 0.05. Models were run on 168 gastric samples genome-wide. **(b)** Venn diagram shows the overlaps among the hotspots detected by models defined in **Figure** **1 (e)**. Models were run on 168 gastric samples in CTCF binding sites only.


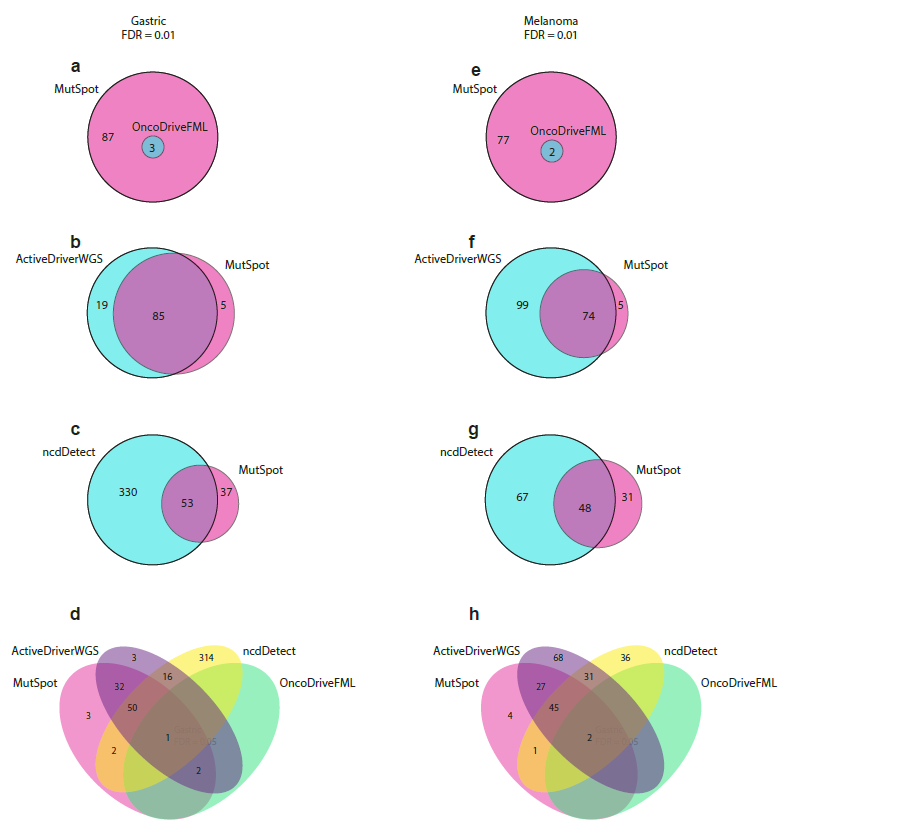


**Supplementary Figure 5.** Overlaps among hotspots identified by MutSpot and other tools adapted for hotspot identification. Venn diagrams showing the overlap between hotspots detected by MutSpot and **(a)** OncoDriveFML, **(b)** ActiveDriverWGS, and **(c)** ncdDetect2 from 168 gastric tumors at FDR of 0.01. **(d)** Venn diagram showing the overlaps of all four methods on the gastric cancer cohort. Venn diagrams showing the overlap between hotspots detected by MutSpot and **(e)** OncoDriveFML, **(f)** ActiveDriverWGS, and **(g)** ncdDetect from 70 melanoma tumors at FDR of 0.01. **(h)** Venn diagram showing the overlaps of all four methods on the melanoma cohort.

**Supplementary Figure 6.** Relationship between number of sampled sites and model fit. LASSO feature selection was performed using 100,000, 200,000, 500,000, 1,000,000, 1,500,000, 2,000,000 (default), 3,000,000 and 4,000,000 sampled sites in the gastric and melanoma cohorts. LASSO feature selection was performed using 12,000, 24,000, 36,000, 48,000 and 60,000 sampled sites in the T-ALL cohort. MacFadden’s pseudo-R2 of the fitted model in each experiment for **(a)** gastric, **(b)** melanoma, and **(c)** T-ALL cohorts. Number of epigenetic (red) and sequence (black) features selected in each experiment for **(d)** gastric, **(e)** melanoma, and **(f)** T-ALL cohorts. Heatmaps showing the recurrence frequency of each feature from 100 bootstraps for **(g)** gastric, **(h)** melanoma, and **(i)** T-ALL cohorts. The LASSO stability threshold was set at 1, 1, and 0.9 for sequence features and 0.75, 0.98, and 0.65 for epigenetic features for the gastric, melanoma and T-ALL cohorts respectively. We increased the selection threshold for the gastric and melanoma cohorts to keep the number of features computationally tractable, and decreased the selection threshold for the T-ALL cohort as the number of mutations in the cohort is underpowered for optimal feature selection.

**Supplementary Figure 7** Effect of window size and minimum recurrence on compute time and number of hotspots detected. MutSpot was run on 168 gastric tumors while varying parameters: *hotspot.size* and *min.count*. Blue axis and blue bar graph shows the number of significant hotspots detected at FDR 0.01. Red axis and red line graph shows the run time of the prediction step. **(a)** *hotspot.size=21* while varying *min.count* from 2-4 on genome-wide analysis. **(b)** *min.count*=3 while varying *hotspot.size*=21,31,41 on genome-wide analysis. **(c)** *hotspot.size=21* while varying *min.count* from 2-4 on CBS-specific analysis. **(d)** *min.count*=3 while varying *hotspot.size*=21,31,41 on CBS-specific analysis.

**Supplementary Figure 8** Time and memory usage of MutSpot when applied to 200, 500 and 1000 cancer whole genomes using **(a)** 4 cores and **(b)** 6 cores.
